# School Meal Programs in Africa: Regional Results From the 2019 Global Survey of School Meal Programs

**DOI:** 10.3389/fpubh.2022.871866

**Published:** 2022-05-26

**Authors:** Ayala Wineman, Moses C. Ekwueme, Liliane Bigayimpunzi, Alice Martin-Daihirou, Eth Ludmilla de Gois V. N. Rodrigues, Priscilia Etuge, Yale Warner, Heidi Kessler, Arlene Mitchell

**Affiliations:** ^1^Global Child Nutrition Foundation, Seattle, WA, United States; ^2^Department of Agricultural, Food, and Resource Economics, Michigan State University, East Lansing, MI, United States; ^3^Hubert Department of Global Health, Rollins School of Public Health, Emory University, Atlanta, GA, United States; ^4^International Initiative for Impact Evaluation (3ie), London, United Kingdom; ^5^Department of Fisheries and Aquatic Sciences, University of Buea, Buea, Cameroon

**Keywords:** Africa, agriculture, education, health, nutrition, school feeding, school meal programs, social protection

## Abstract

**Introduction:**

School meal programs operate throughout Africa, serving as a social safety net and aiming to improve children's nutrition, influence their dietary choices, and strengthen the agrifood economy through local procurement. Despite their rapid expansion in the past decade, there has been no systematic effort to comprehensively document school feeding activities across the continent.

**Methods:**

Detailed information on school feeding activities in each country was captured in the Global Survey of School Meal Programs©, which launched in 2019. An invitation to participate was extended to each government, which appointed a national-level respondent to gather information on every large-scale school meal program in the country.

**Results:**

Forty-one countries in Africa (38 in sub-Saharan Africa) responded to the survey in 2019 with information on 68 large-scale programs that together reached 60.1 million children. Across these countries, the aggregate school feeding budget was USD 1.3 billion. Diversity in school meal programs is evident across regions, country income levels, and levels of national commitment. Coverage rates tended to be highest in southern Africa, in countries with school feeding as a line item in the national budget, and in countries with the greatest domestic share of the school feeding budget. Diversity in the school menu tended to be greatest in programs that sourced food through domestic purchase rather than relying on foreign in-kind donations. To address micronutrient malnutrition, about two-thirds of the programs served fortified foods, and one-quarter included micronutrient supplements. Even as rates of overweight/obesity are rising among African school children, just 10% of school meal programs identified its prevention as an objective.

**Conclusion:**

The extent to which school meal programs in Africa are supported with domestic funding reflects a dramatic shift in favor of national ownership and domestic food procurement. At the same time, programs have grappled with inadequate and unpredictable budgets and challenges related to supply chains and logistics—impediments that need to be addressed if these programs are to achieve their objectives. Overall, the survey results underscore the important position of school meal programs within African food systems and their potential (if well-designed) to sustainably improve food security, child health, and nutrition.

## Introduction

School meal programs—through which students are provided with meals, snacks, or take-home rations—comprise one of the most widespread safety nets in the world, reaching an estimated 388 million children (1) and operating in a greater number of countries than any other safety net program (2). For many children, particularly those in low-income settings, the food served in schools represents their only regular meal of the day, making school meal programs relevant to achieving the second Sustainable Development Goal (SDG) of ending hunger. The past decade has seen a rapid expansion of school meal programs in Africa, with the number of children who benefit growing by 71% between 2013 and 2019 (3, 4), and African governments have increasingly exhibited support for school feeding through their budget allocations and policy frameworks (4, 5).

School meal programs are intended to address multiple cross-sectoral objectives. They aim to enhance access to education by reducing barriers to school enrollment, raising attendance and retention, increasing students' ability to concentrate during the school day, and improving learning outcomes (3). They also aim to reduce the gender gap in education by addressing barriers to schooling that are particularly salient for girls (6, 7). By targeting children from low-income households, school meal programs additionally serve as a social safety net (4, 8, 9). They address objectives related to health and nutrition by reducing hunger and improving children's micronutrient status with diverse menus and food fortification, and, particularly in high income settings, school meal programs are often designed to model healthy eating habits and influence children's food choices (10).

Along these lines, evidence has accumulated regarding the positive impacts of school meal programs, with effects often mediated by variations in program design. Many studies have documented a positive impact on school enrollment, attendance, and retention, particularly where baseline levels of school participation are low (9, 11–13). In-school feeding has been found to have a greater impact on enrollment for girls than for boys (14), though in at least some cases, the persistence of this pattern is contingent on the supplementary provision of take-home rations for girls (6, 12). There is also considerable evidence of the impact of school feeding on children's cognitive performance and educational achievement (11, 14). In terms of health and nutrition, there is evidence of positive outcomes for children's height and weight (13) and micronutrient status, such as hemoglobin concentration/anemia and vitamin A status (15, 16). A recent analysis of school meal programs took account of impacts across multiple sectors and arrived at a benefit-cost ratio of between 7 and 35 (17), attesting to the numerous benefits generated by such programs.

In recent years, home-grown school feeding (HGSF) has increasingly gained traction. HGSF programs incorporate the procurement of locally grown food into the design of school meal programs with the intent to promote local economic development and agricultural transformation. By meeting the schools' demand for food with that supplied by smallholder farmers, these programs aim to foster a new market for farm output and create jobs all along the food value chain (4, 9, 18, 19). Local procurement is further employed to address health and nutrition objectives by ensuring that school menus contain a variety of nutritious foods (10, 16, 20–22). However, as HGSF programs are a more recent innovation, there is limited evidence regarding their impacts on agricultural and local economic development (3).

Reflecting their mani-fold objectives, school meal programs encompass a diverse set of designs and implementation arrangements. Programs can vary in the modality through which food is provided, the contents of the menu, the way children are targeted to receive food, the embedding of conditions into the criteria for participation, and the pairing of school meals with other health and nutrition programs, among many other factors. The three main modalities through which food is provided to school children include in-school meals, in-school snacks (such as fortified biscuits, fruits, or milk), and take-home rations given to the students' families, often conditional upon their children maintaining a certain rate of school attendance (3).

In addition to variation in the food items served, school meal programs can vary in their inclusion of fortified foods, biofortified foods, or micronutrient supplements to enhance the nutritional content of the menu. School meals and snacks may also vary in their site of preparation (on school grounds or off-site) and their level of processing, and the programs can vary in their level of centralization, with decisions alternatively made at the central, regional, local, or school levels. Finally, programs may choose to incorporate a wide variety of complementary services, such as deworming treatment, handwashing with soap, or nutrition education, which augment the value of the food provided (7).

Even as school meal programs have grown in scale, scope, and function, the data landscape on school feeding tends to be fragmented, with inconsistent quantity and quality of information across countries and even across different programs within the same country (23). While it is relatively easy to find information on programs implemented by the World Food Program or other international partners, information on nationally owned programs (i.e., those managed by governments, either alone or with support from development partners) can be quite scarce—though the latter are substantial in scale and geographic reach. Furthermore, information is not collected and published regularly, making it difficult to compare school feeding operations across different settings or discern trends over time.

The disarray in the school feeding data landscape prompted Bundy et al. (24), pp. 94–95 to call for “a database on school feeding programs that describes the coverage and functioning of programs globally… [in order] to estimate, for example, the global population served by school feeding programs, the gaps in coverage, the costs of different programs, the regularity of program functioning, or the popularity of different modalities.” In response to this call, the Global Survey of School Meal Programs © was launched in 2019, capturing information on the scope and nature of school feeding activities in each country in a consistent, comprehensive, and recurring manner.

This paper presents results for the 41 African countries that responded to the Global Survey of School Meal Programs in 2019. Results are used to estimate the scale, coverage, and budgets of school meal programs in Africa; characterize the programs and their beneficiaries; analyze the food baskets provided and food sources accessed; assess various health and nutrition aspects of the programs; and comment on the enabling environment around school feeding.

## Materials and Methods

### Data

The 2019 Global Survey of School Meal Programs collected information on the existence of school meal programs in each country. The survey was reviewed by the University of Washington Institutional Review Board and was deemed to be exempt from consent procedures, as this data collection exercise did not constitute human subject research. The survey was based on the United Nations listing of 193 countries plus Palestine, which has observer status at the U.N., and the survey's reference period was the most recently completed school year, which was 2017/2018 for most countries in Africa.

The survey captured detailed information on the number and characteristics of beneficiaries; the avenues through which school meal programs procured and distributed food; the extent and nature of government involvement with school feeding; job creation in school meal programs and engagement with farmers and the private sector; and related health and sanitation topics. While some information was collected at the country level, most information was collected at the level of each large-scale school feeding program. In the context of this survey, this is defined as a program that is managed and/or administered by the national government, by regional or local governments, or by a non-governmental entity in coordination with the national government, or one that reaches a substantial proportion of students in the country or covers a substantial geography.

Data collection took place throughout 2019, when the survey team reached out to national governments to secure their cooperation. Each government designated a “focal point,” an individual who was knowledgeable about school feeding activities in the country and/or could gather needed information to complete the survey. To ensure a consistent understanding of terminology, the survey was accompanied by a detailed glossary of terms used in the questionnaire. The data set and further details on the data collection process can be accessed through the Global Child Nutrition Foundation (23).

**Figure 1:**
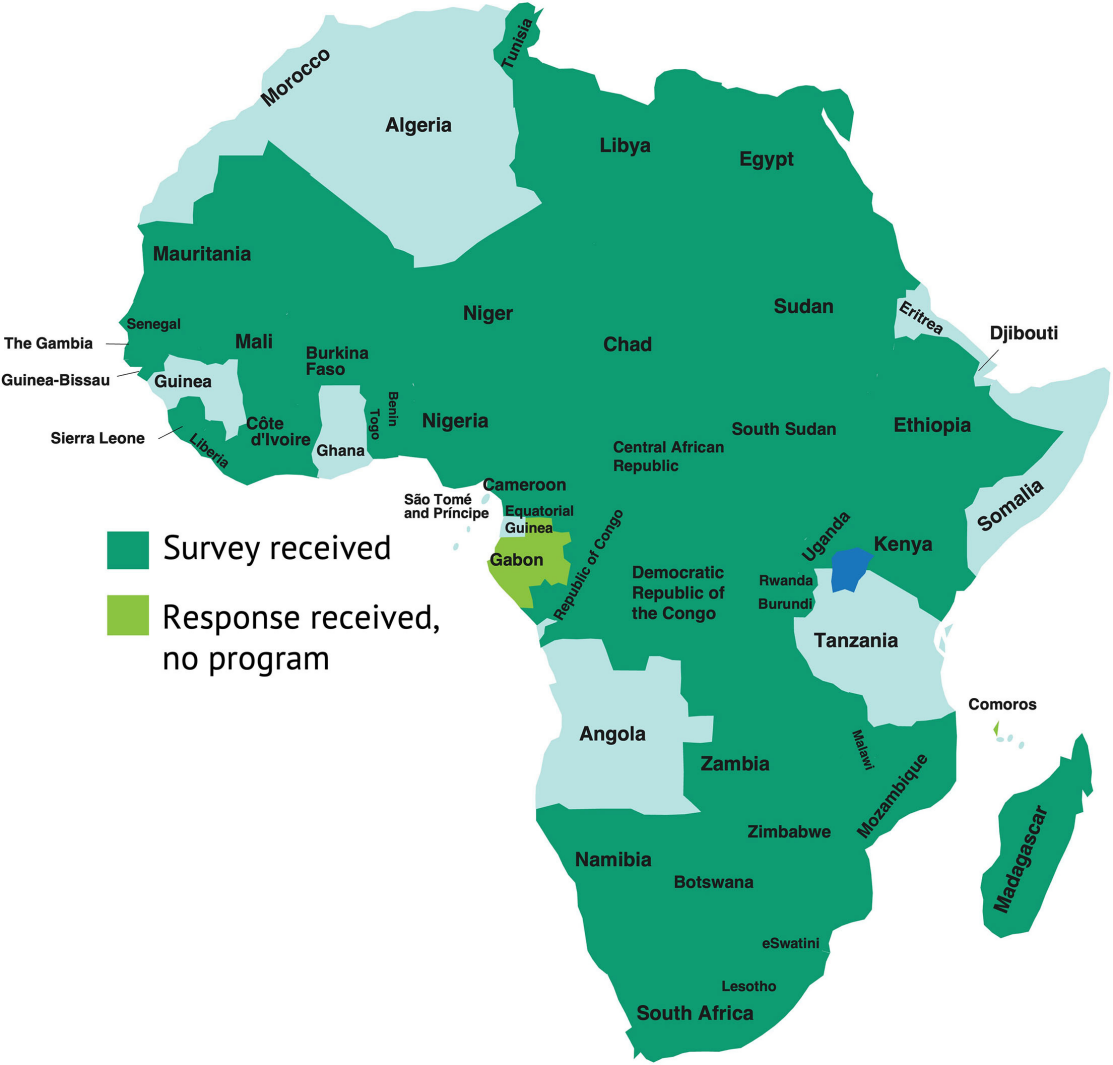
Survey response status in Africa.

Of the 54 countries in Africa, 41 countries (38 in sub-Saharan Africa (SSA)) responded to the survey in 2019 (Figure 1). These countries were Benin, Botswana, Burkina Faso, Burundi, Cameroon, Central African Republic, Chad, Comoros, Congo, Côte d'Ivoire, Egypt, Ethiopia, Gabon, The Gambia, Guinea-Bissau, Kenya, Lesotho, Liberia, Libya, Madagascar, Malawi, Mali, Mauritania, Mozambique, Namibia, Niger, Nigeria, Republic of Congo, Rwanda, São Tomé and Príncipe, Senegal, Sierra Leone, South Africa, South Sudan, Sudan, Togo, Tunisia, Uganda, Zambia, Zimbabwe, and eSwatini. This equals 76% of the countries in Africa (79% in SSA), which together held approximately 82% of the continent's population as of 2017 (86% in SSA). Two countries (Comoros and Gabon) reported that they had no large-scale school feeding activities, while the others together provided information on 68 programs.

As of 2017, half of the countries in Africa were categorized as low income; one-third were considered lower middle-income; eight countries were upper middle-income; and just one country was high income (25). The African response rate to the 2019 Global Survey of School Meal Programs tended to decline with rising wealth levels, such that 85% of low-income countries, 72% of lower middle-income countries, and 63% of upper middle-income countries responded to the survey. The sole high-income country did not respond. Summary statistics in this paper reflect only the sample of respondent countries, and hence most accurately capture conditions in low-income and lower middle-income settings within Africa.

### Variables and Methods

The survey results are used to construct key indicators related to school feeding, several of which merit explanation. First, the school feeding coverage rate for a given country or region is defined in this analysis as the share of primary and secondary school-age children (usually ages 6 through 18) that received food through school meal programs. The denominator in this indicator is therefore inclusive of both enrolled students and out-of-school children/youths. Second, budgets for school meal programs have been converted to United States dollars (USD) using an exchange rate that correlates to the timing of the school year in each country. However, these monetary values have not been standardized to account for differences in the length of school year or number of school feeding days, which can vary across countries and programs. Third, some indicators, such as the share of food from local sources, reference “local” settings. “Local” refers to an administrative level more localized than the region/state/province level, hence at the district, county, municipality/town, or community level. Fourth, the survey did not ask focal points to categorize programs as being “home-grown” or not, given the ambiguity in this delineation. However, some programs are named this way, and many respondents used the term in their narrative accounts of school feeding in their countries. The discussion below therefore maintains this language. Fifth, the survey collected information on food sources and avenues of procurement. In the analysis below, school meal programs are alternately classified as either relying on domestic purchase (drawing at least 70% of food through purchase and purchasing only from domestic sources), relying on foreign in-kind donations (drawing at least 70% of food through in-kind donations, at least some of which came from faraway countries), or relying on neither.

These variables related to school feeding are analyzed in a descriptive manner using Stata (version 16.1), alternately reporting average values across countries, aggregate values across Africa or Africa's subregions, or average values (or percentages) across school meal programs. Correlations between two variables are sometimes also calculated using a simple linear regression.

## Results

### Coverage of School Meal Programs and Characteristics of Beneficiaries

Across the 39 African countries with school feeding, 59% had just one school meal program, 18% had two programs, 13% had three programs, and 10% had four programs in operation. In these countries, an estimated 60,053,496 children of all ages received food through school meal programs. The three countries with the greatest absolute numbers were Egypt (11.52 million), Nigeria (9.83 million), and South Africa (8.95 million); the rate at which Nigeria's national school meal program was scaled up is particularly noteworthy, as it was newly launched in 2016.

Across the 41 African countries that submitted a survey response, the average school feeding coverage rate was 23%. Similarly, when aggregating across countries (i.e., when summing populations rather than calculating a cross-country average), 21% of school-age children received food through their schools (Table 1). Coverage increased with wealth, rising from 15% in low-income countries to 24% in lower middle-income countries and 67% in upper middle-income countries.

**Table 1:** School feeding coverage rates (percent of school-age children receiving food).

		**Coverage rate (%)**	
		**Cross-country average**	**Total (aggregated across countries)**
**A. Primary and secondary school age**			
	All countries	23	21
Income group	Low-income	17	15
	Lower middle-income	26	24
	Upper middle-income	46	67
Region	Central	11	3
	Eastern	11	12
	Northern	21	40
	Southern	50	42
	Western	19	22
**B. Primary school age**			
	All countries	33	30
Income group	Low-income	24	20
	Lower middle-income	38	37
	Upper middle-income	61	73
Region	Central	19	4
	Eastern	14	17
	Northern	36	71
	Southern	63	45
	Western	33	35
**C. Secondary school age**			
	All countries	7	6
Income group	Low-income	4	3
	Lower middle-income	8	2
	Upper middle-income	16	57
Region	Central	0	0
	Eastern	5	4
	Northern	4	4
	Southern	20	24
	Western	4	2

Coverage rates also varied across regions within Africa, ranging from 3% in central Africa to 42% in southern Africa. Eight countries had school feeding operations that reached at least half of their primary and secondary school age children, including Namibia (50%), Burkina Faso (52%), São Tomé and Príncipe (53%), Lesotho (56%), Botswana (62%), Zimbabwe (67%), South Africa (72%), and eSwatini (85%).

The survey results revealed a striking correlation between coverage rates and having school feeding as a national budget line item. Across the 13 countries with no line item, 15% of primary and secondary school-age children received food through their schools, while across the 28 countries with a line item, this value was 25%. Countries in central or eastern Africa were least likely to report school feeding as a line item.

All African countries with school feeding programs provided food to those in primary school, with seven countries reporting that they reached at least 80% of their enrolled primary school students, including Botswana, Burkina Faso, Egypt, Lesotho, São Tomé and Príncipe, Sierra Leone, and eSwatini. Over half (56%) of the countries also provided food to pre-school students; 44% reached students in secondary school; and two countries (Burkina Faso and Madagascar) reached some students in vocational/trade schools. It follows that coverage rates for primary school-age children tended to be higher than for other ages: Across all countries, 30% of primary school-age children received some food through their schools, while this value is just 6% for those of secondary school-age.

Across the 68 school meals programs, 63% provided gender-disaggregated student numbers, usually reporting a roughly equal gender breakdown of beneficiaries. Many of the school meal programs were targeted geographically, serving all schools within a given area that was selected based on the prevalence of poverty/food insecurity and rates of school enrollment/attendance. At the same time, three quarters of programs with take-home rations targeted these with consideration of individual characteristics, such as the students' gender or poverty status.

### Characteristics and Components of the School Meal Programs

School meal programs in Africa exhibited a range of objectives. All were designed to meet educational goals, 88% aimed to meet nutritional and/or health goals, and 81% served as a social safety net, ensuring food access for poor or vulnerable children. It was less common (at 46%) for programs to report agricultural objectives, and just 10% of the programs in Africa explicitly aimed to prevent obesity.

In-school meals were the most common modality through which food was provided, with 94% of programs serving meals in schools, 12% serving snacks, and 26% providing take-home rations. It was common for programs to pair meals/snacks with take-home rations; in fact, there were no programs that *only* provided take-home rations. In-school meals were served (or at least intended to be served) 5 or 6 times per week in 92% of the programs and 2 times per week in another 8%. Take-home rations were provided less frequently, often at monthly intervals or at other frequencies, such as quarterly, biannually, or during the lean (hunger) season. Lunch was part of school meal programs in 90% of the countries, while breakfast was served in 31% of the countries. Only programs in Niger and Tunisia served an evening meal, generally in the context of public boarding schools.

**Figure 2:**
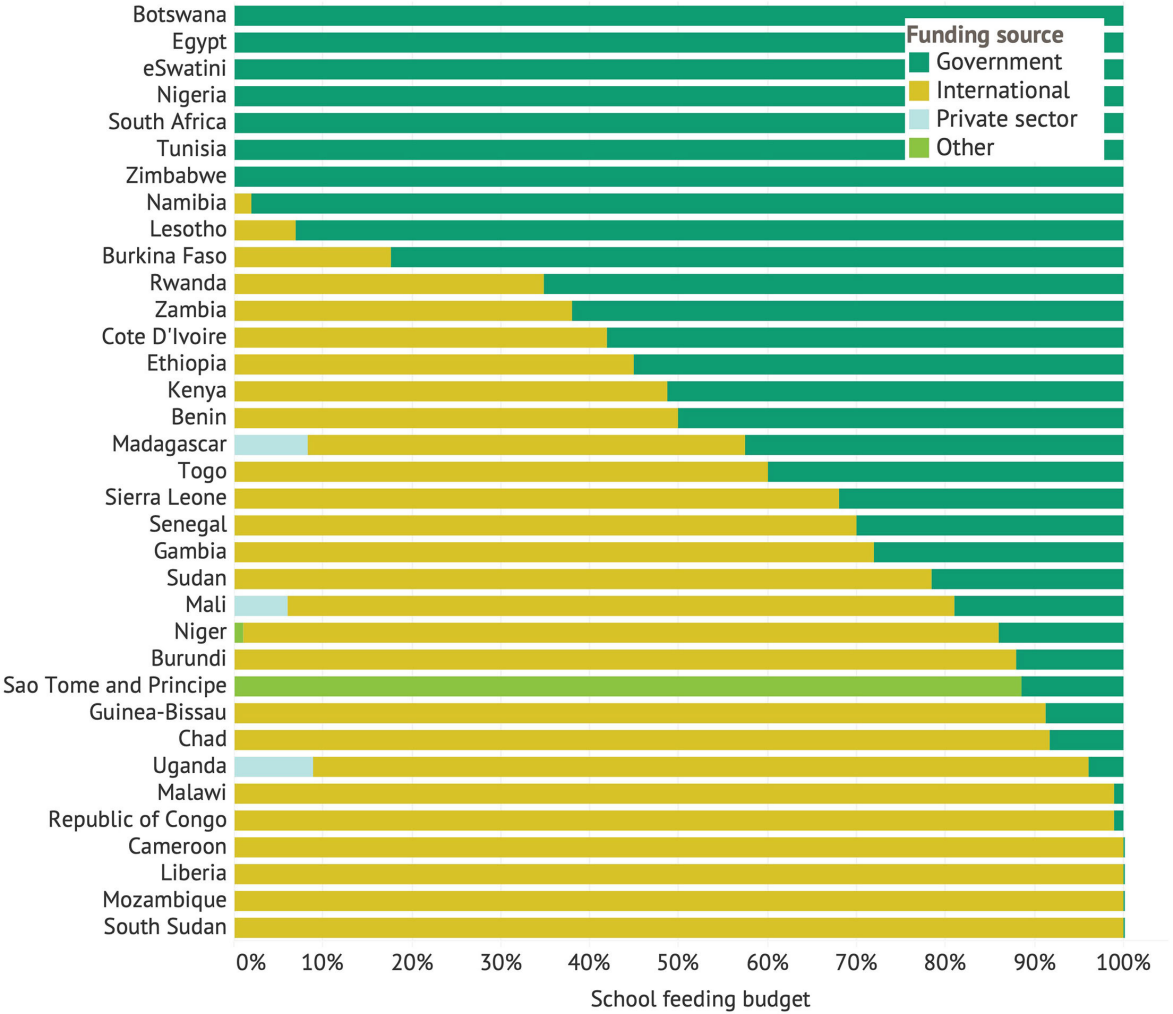
Sources of funding for school meal programs in Africa. Note: Information on the school feeding budget was not available for the Central African Republic, Congo, Libya, or Mauritania.

Across the 41 African countries that responded to the survey, the total school feeding budget summed to USD $1,318,904,945 for the most recently completed school year. In aggregate, the continent spent $22 per year per beneficiary child. As expected, this value increased with rising wealth (from $16 in low-income countries to $56 in upper middle-income countries) and varied across regions (ranging from $7 in northern Africa to $34 in southern Africa). Across countries, the average share contributed by government was 45%, the average share from international sources was 51%, and the private sector or other sources provided the rest. While 14 countries contributed less than one quarter of their school feeding budget, 10 countries contributed over three quarters of the budget (Figure 2). Furthermore, when aggregating across countries, 80% of the total (summed) budget for school feeding on the continent came from African governments.

The survey results reveal a positive correlation between the national school feeding coverage rate and the government share of the school feeding budget (Coefficient in a simple linear regression = 0.375, *P* < 0.001)—a correlation that remains statistically significant even when controlling for total budget size and/or budget per beneficiary child. Though countries with the greatest domestic funding share tended to be clustered in southern Africa, some outliers demonstrate that this goes beyond regional variation. Burkina Faso, for example, had a domestic funding share of 82% and a coverage rate of 52%. Domestic funding of school meal programs is a strong driver of the coverage and sustainability of school feeding.

### Food Basket and Food Sources

The food basket contents in African school feeding programs are presented in Table 2. Grains/cereals were provided in almost all programs (at 98.5%), as were oil (90%), legumes (87%), and salt (78%). Green leafy vegetables, other vegetables, fish, and tubers were provided in 25–50% of cases, while it was uncommon for poultry, eggs, and dairy products to be included. Dairy products were more commonly included on the menu in upper middle-income countries (at 50% of programs), compared to lower middle-income countries or low-income countries (at 18 and 15% of programs, respectively). In their responses to open-ended questions, the focal points (national survey respondents) often celebrated the inclusion of new food items, as in Burundi, which had recently introduced farm-sourced dairy products to schools, or South Africa, which had recently added sardines to the school meal menu.

**Table 2:** Food items served in school meal programs.

**Category**	**% of programs**
Grains/cereals	99
Oil	90
Legumes, nuts	87
Salt	78
Green leafy vegetables	37
Other vegetables	31
Fish	28
Roots/tubers	27
Sugar	24
Meat	21
Fruits	19
Dairy products	18
Eggs	15
Poultry	9

Of 14 broad food categories (eggs, dairy, fruit, etc.), the food baskets of school meal programs contained an average of just 5.7 categories. There was some regional variation, with the average number of food categories highest in southern Africa (at 6.8) and lowest in eastern and northern Africa (at 4.5 each). The food basket contents also tended to vary by the modality through which children received food. On average, in-school meals contained foods from 7.4 categories, in-school snacks contained 1.5 categories (often grains in the form of biscuits or porridge), and take-home rations contained 2.1 categories (often grains and oil). Countries in Africa that reported having a national policy related to nutrition in school feeding programs tended to have more diverse school meal menus (with an average of 7.3 food categories) than those with no such policy (average = 5.5 categories).

The most common avenue through which school meal programs in Africa procured food was through domestic purchase, with 83% of programs accessing at least some food through this avenue. This was followed by receipt of in-kind donations from within the country (in 50% of programs) and in-kind donations from other countries (in 47% of programs). Foreign purchases were the least common procurement choice (in 29% of programs).

In-kind donations from foreign countries tended to come from faraway countries, i.e., not in the same economic community or “neighborhood.” The World Food Program provided the food in 66% of the programs that received foreign in-kind donations, while in-kind donations from domestic sources tended to come from within the local community, often taking the form of parents supplying ingredients, such as condiments, to their children's schools. In 11% of programs that received in-kind donations from within the country, this came from private businesses. For example, the National School Nutrition Program (NSNP) in South Africa provided school lunches but was supplemented by private sector (in-kind) support to also provide some breakfasts.

The domestic purchase of supplies was celebrated in many countries, such as Namibia and Nigeria. Processors in Egypt produced the baked goods for school snacks, while processors in Malawi produced the corn-soy blend served in schools. Among the programs that purchased any food, 77% procured at least some from within the local community. Examples included the National School Feeding Program of Mali, the Home-Grown School Feeding Program in Ethiopia, the National School Feeding Program in Burundi, and the Mary's Meals Program in Malawi. A number of programs reported on recent, ongoing, or anticipated transitions toward a HGSF approach to food procurement. Thus, while the school meal program in Guinea-Bissau incorporated the purchase of local agricultural products in 2014, the shift in Liberia has been more recent, and the Namibian School Feeding Program is just now introducing a HGSF model.

**Figure 3:**
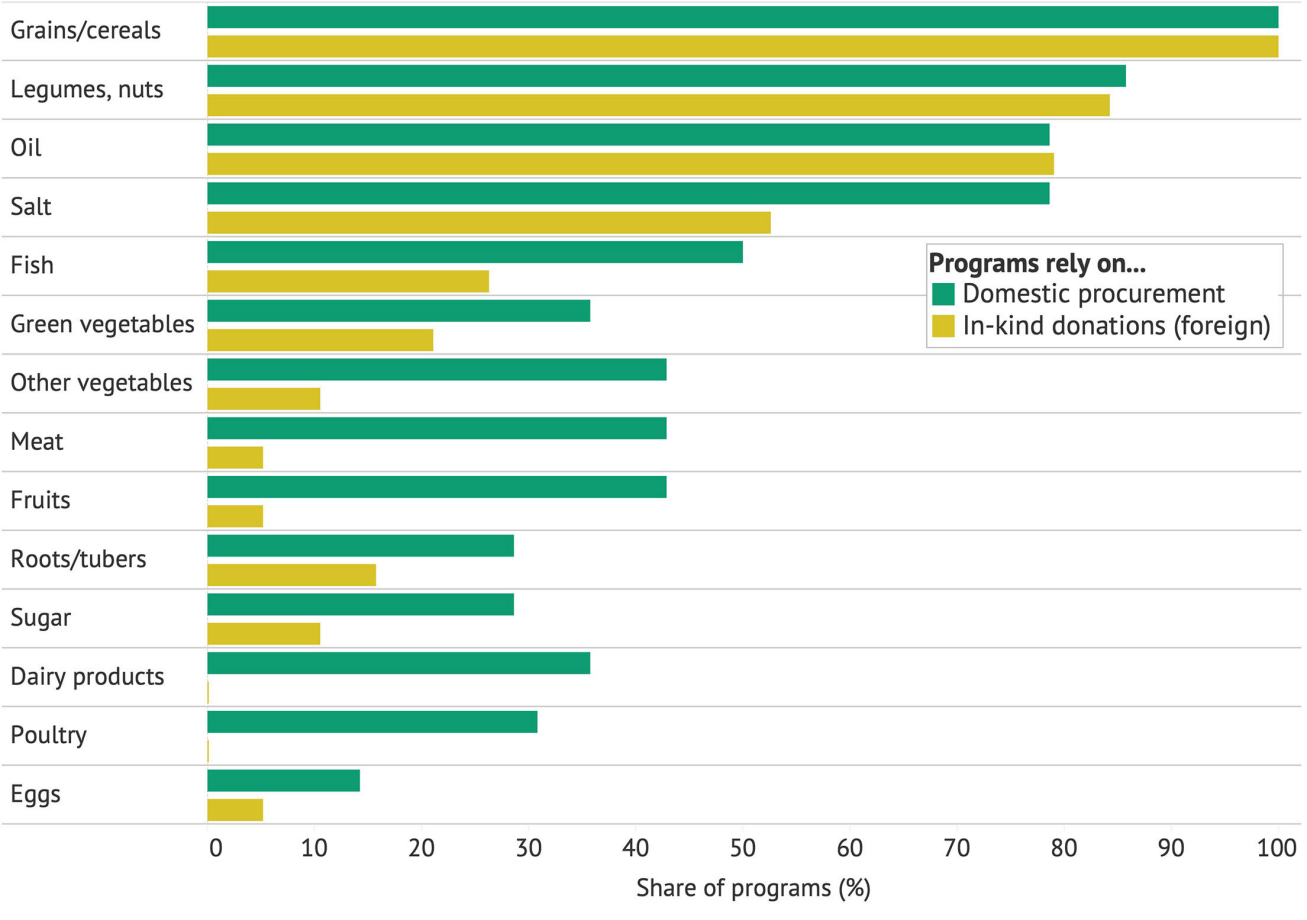
Food basket contents and avenue of food procurement in school meal programs.

Food basket contents tended to be correlated with the primary avenue through which food was procured. While 20 of the programs in Africa relied primarily on domestic purchase, 12 programs relied on foreign in-kind donations (see variable definitions in section 2). Figure 3 shows the typical food basket contents across these two categories. While most programs in both categories included grains and oil, it was far more common for the menu in programs that relied on domestic purchase to include fish (50%), meat (43%), fruit (43%), green leafy vegetables (36%), poultry (31%), and eggs (15%), among other items. (Programs that received some in-kind donations, but did not rely on them, tended to have menus in between the two extremes depicted.) It is evident that a reliance on foreign food donations is correlated with less diverse menus. Interestingly, there is no correlation between a program's budget per child and the number of food categories served (Coefficient in a simple linear regression = −0.004, *P* = 0.267). In other words, the source of food seems to be more important than the budget for menu diversity.

### Health and Nutrition

Beyond the inclusion of different food categories, school meal programs have a number of tools available to enhance their nutrition status. Sixty-eight percent of the programs in Africa served fortified foods, such as oil, salt, grains/cereals, corn-soy blend, or biscuits fortified with vitamin A, iodine, and iron (among other micronutrients). At 30%, it was less common for programs to include micronutrient supplements either added to the food or provided directly to the children. A few programs also served biofortified foods, such as the vitamin A-rich orange flesh sweet potatoes used in The Gambia, Malawi, Mozambique, and Nigeria.

Noting that school feeding is but one component of school health, and that the effects of school meals are mediated by other aspects of health, the survey also gathered information on complementary programs and services offered in schools. It was common for school meal programs to be paired with other health or hygiene services/programs (Table 3). All school meal programs in Africa incorporated handwashing into the school feeding activities. The provision of potable drinking water was the next most common accompaniment to school meals (in 86% of programs), followed by deworming treatment (in 78% of programs). Note that worm infections can result in iron deficiency, such that deworming enhances the effectiveness of school meal programs by facilitating the absorption of iron. Other services, such as eye testing or menstrual hygiene programs, were offered less often.

**Table 3:** Complementary services and education programs.

**Services**	**% of programs**	**Education programs**	**% of programs**
Handwashing	100	Hygiene	92
Drinking water	86	Nutrition	89
Deworming	78	School gardens	84
Water purification	34	Health	81
Menstrual hygiene	32	HIV prevention	55
Weight measurement	17	Physical education	53
Height measurement	15	Food and agriculture	52
Dental testing	12	Reproductive health	40
Eye testing	6		
Hearing testing	6		

It is similarly common to find complementary education programs offered as part of the school feeding package. Thus, 89% of programs offered nutrition education, and 84% were paired with school gardens. Among those that included school gardens, the garden products were consumed by students in 98% of the cases and were also sold in 48% of the cases. In Tunisia, a common arrangement was for one third of garden production to be used in the school meals program, while the remainder was sold.

### Enabling Environment for School Feeding

A robust enabling environment in terms of political commitment, a strong policy and regulatory framework, and supportive infrastructure is necessary for school meal programs to thrive. More specifically, a national school feeding policy can help solidify a country's commitment and clarify its objectives and strategies for school feeding, and nutrition standards can likewise sharpen attention to nutrition objectives (16). About three-quarters (74%) of the African countries with school feeding had a national school feeding policy, law, or standard. It was also somewhat common (at 62%) for countries to have a policy related to school feeding regarding nutrition, and 44% had a policy regarding food safety. Just over half (51%) had a policy regarding agriculture linked to school feeding. Note that 47% reported some direct involvement of farmers in school meal operations, often with the intent to bolster the rural economy through local food purchases.

A monitoring and evaluation (M&E) system is critical for oversight and quality assurance in school meal programs, and a country-wide system for monitoring school feeding programs was reported in 87% of the countries in Africa. Namibia maintains a system of data capture through the Namibian School Feeding Information System (NaSIS), though consistency in data entry remains a challenge.

Infrastructure also plays a key role in school meal programs. One half of the African countries reported that all or most schools had clean water, while 15% reported that few or no schools had clean water. At the same time, all or most schools had cafeterias or other dedicated eating spaces in just 9% of the countries, while 59% reported that very few or no schools had cafeterias. One half of the countries reported that very few or no schools had electricity; this has implications for the ability of schools to refrigerate or preserve food items.

Most school meals or snacks in Africa were prepared on school grounds. Among the 88% of programs that used charcoal/wood stoves in school kitchens, students were expected to provide fuel in 45% of the cases. Challenges related to deforestation, exacerbated by the use of firewood in school meal preparation, and to finding energy for cooking were highlighted in Burundi, Malawi, and Niger. In addition, 7% of programs brought in food from off-site private kitchens. Just 1.5% of programs only served foods that were purchased in processed form and required no preparation.

### Challenges

Though the survey focal points (respondents) could enumerate many successes and positive developments related to school feeding in Africa, the associated challenges were also abundant. Inadequate and unpredictable budgets were reported in many countries, including Côte d'Ivoire, Liberia, and Niger. In fact, funding was considered “adequate” in just 38% of the programs. For example, when the World Food Program ended its support for Zambia's Home-Grown School Feeding program, the remaining government budget was deemed inadequate. Countries that lacked a budget line for their school feeding programs (including Cameroon, Guinea-Bissau, and Mozambique) particularly noted this as a problem.

Difficulties related to supply chains and logistics were also acknowledged. Food losses in storage or in transit to schools were acknowledged in Kenya, with food sometimes being condemned by public health officials due to spoilage. School access was limited during the rainy season in Benin and Sudan, particularly in regions of poor road quality. Respondents for Cameroon, Mali, and Niger also reported that parts of the country were difficult to access due to conflict and socio-political upheaval, or that population displacements caused by security crises disrupted school feeding programs.

Insufficient or inadequate human resources were also noted in Botswana, Guinea-Bissau, Liberia, Madagascar, and Sierra Leone. Among other challenges, frequent turnover of school feeding personnel resulted in inefficiencies and the allocation of scarce resources toward redundant training. Several countries acknowledged weaknesses in their monitoring and evaluation systems or found that that completing the survey was difficult due to a lack of data stemming from poor record-keeping.

Finally, despite the widespread enthusiasm for HGSF, challenges around local procurement were common. Such procurement was particularly challenging in Kenya's arid regions, which is where the Home-Grown School Meals Program operated. Similarly, the School Feeding Program in Mauritania specifically operated in food insecure and vulnerable areas with limited agricultural production. In Liberia and Malawi, limited production even at the national level presented an obstacle for school meal programs.

## Discussion

Results from the 2019 Global Survey of School Meal Programs confirm school feeding's place in African food systems, with meal programs present in 95% of the countries and reaching 30% of all children of primary school age. Such programs have grown in popularity in the past decade and are increasingly employed to serve as a safety net, improve children's nutrition, meet education goals, and bolster rural economies. The survey results reveal great diversity in school meal programs across Africa, with variation evident across regions, income levels, and levels of national commitment. Several themes from the survey results deserve mention.

The extent to which school meal programs in Africa are supported with domestic funding seems to reflect a dramatic shift in favor of national ownership (3, 5). This has profound implications for the sustainability of these programs, the coverage achieved, and even the diversity of food provided. The survey results indicated that there was a positive correlation between the government share of the budget and the national school feeding coverage rate. Moreover, countries with a budget line for school feeding were more secure in their funding and were found to reach a greater share of children. Altogether, this underscores the importance of government commitment to school feeding, with policy implications for efforts to increase school feeding in Africa.

At the same time, for programs operating in less supportive environments, a key theme was the tremendous stress of unreliable funding, which was regarded as inadequate in 62% of programs. This inhibited the programs from reaching their targets and scaling up further. The positive correlation between a country's wealth level and its school feeding coverage rate starkly demonstrates how school feeding tends to be scarcest precisely where needs are greatest (11, 23). Efforts are needed to stabilize and expand budgets for school feeding in the African countries where food insecurity and child malnutrition are likely to be highest.

One particularly encouraging takeaway from the survey was the enthusiastic embrace of HGSF approaches to food procurement, even if this still seems to characterize a minority of Africa's school meal programs. The HGSF concept was first introduced in Africa in 2003, when the New Partnership for Africa's Development (NEPAD) orchestrated HGSF pledges from 11 countries (18). The survey results indicated that a reliance on domestic purchase rather than in-kind foreign food donations was correlated with more diverse school meal menus. In narrative accounts, the focal points (survey respondents) expressed strong support for HGSF, with a projection that HGSF models would be scaled up in coming years. Nevertheless, the survey also exposed some ambiguity regarding the definitional criteria of HGSF, with programs referred to as HGSF when they sourced food from local markets near individual schools, and also when they implemented a fully centralized approach to procurement and distribution. Clearer definitions and a typology of HGSF programs would shed light on this situation and inform research on the optimal HGSF program design—a topic with limited evidence to date (3).

Another salient finding from the survey was the limited attention given to overweight/obesity in school meal programs in Africa, with just 10% identifying overweight/obesity prevention as an objective. As Africa becomes increasingly urbanized, it has undergone a nutrition transition in favor of purchased and processed foods, with limited consumption of fruits and vegetables but high levels of sugar intake (26). Not surprisingly, overweight and obesity have grown increasingly prevalent among African school children, especially those attending urban and/or private schools (27, 28). The establishment of healthy eating habits among children and adolescents is imperative for reducing their risk of non-communicable diseases in later years (29), and school meal programs could play a role in this realm (10). Nevertheless, green leafy vegetables, other vegetables, and fruits were served in just 37, 31, and 19% of programs, respectively. There is clearly scope for allocating greater attention to the prevention or mitigation of overweight/obesity in the design of Africa's school meal programs.

A final lesson from the survey is that African countries can learn a great deal from one another in terms of strategies for scaling up school meal programs, drawing political support and financial commitment, diversifying menus, and building local capacities for program oversight and implementation. Peer-to-peer learning among individuals in different countries who share similar school feeding responsibilities, challenges, and motivations can be particularly powerful (30). The development of a standardized database on school feeding, resulting from the Global Survey of School Meal Programs, along with the shared vocabulary offered in the survey glossary, should also facilitate learning across countries. Data from the second round of the survey, conducted in 2021 and capturing the responses of school meal programs to the COVID-19 pandemic, is forthcoming.

Several limitations of the survey should be acknowledged: First, the data are self-reported and may be influenced by various factors. Governments may aim to issue a positive report of indicators that are considered to reflect positively on the country, such as the school feeding coverage rate. At the same time, governments seeking additional funding for school meal programs (whether from domestic or external sources) may be inclined to issue a less positive report to emphasize the need for support. Second, the data from the 2019 survey reflect a snapshot of a school meals landscape that is dynamic and evolving. As noted, the data from 2019 were collected before the COVID-19 pandemic and therefore do not capture the effects of this global crisis.

## Data Availability Statement

The dataset analyzed in this study is based on the 2019 Global Survey of School Meal Programs©. The dataset is available to the public and can be accessed via the Global Child Nutrition Foundation (info@gcnf.org).

## Author Contributions

AM, AW, and HK conceptualized and initiated the study. Data collection was undertaken by AM-D, LB, ME, EG, and PE. AW conducted the data analysis. AW, YW, ME, EG, and PE wrote the first draft of the paper, which was reviewed by AM, LB, HK, and AM-D. All authors contributed to the article and approved the submitted version.

## Funding

Funding for the 2019 and 2021 Global Survey of School Meal Programs © has been provided, in part, by the United States Department of Agriculture through agreement number FX18TA-10960G002.

## Conflict of Interest

The authors declare that the research was conducted in the absence of any commercial or financial relationships that could be construed as a potential conflict of interest.

## Publisher's Note

All claims expressed in this article are solely those of the authors and do not necessarily represent those of their affiliated organizations, or those of the publisher, the editors and the reviewers. Any product that may be evaluated in this article, or claim that may be made by its manufacturer, is not guaranteed or endorsed by the publisher.
